# Nanometer Scale Titanium Surface Texturing Are Detected by Signaling Pathways Involving Transient FAK and Src Activations

**DOI:** 10.1371/journal.pone.0095662

**Published:** 2014-07-07

**Authors:** Willian F. Zambuzzi, Estevam A. Bonfante, Ryo Jimbo, Mariko Hayashi, Martin Andersson, Gutemberg Alves, Esther R. Takamori, Paulo J. Beltrão, Paulo G. Coelho, José M. Granjeiro

**Affiliations:** 1 Departmento de Química e Bioquímica, Instituto de Biociências, Universidade Estadual Paulista - UNESP, Botucatu, São Paulo, Brazil; 2 Faculdade de Odontologia de Bauru, Universidade de São Paulo, Bauru, São Paulo, Brazil; 3 Department of Prosthodontics, Faculty of Odontology, Malmö University, Malmö, Sweden; 4 Department of Chemical and Biological Engineering, Applied Surface Chemistry, Chalmers University of Technology, Gothenburg, Sweden; 5 Department of Cell and Molecular Biology, Institute of Biology, Universidade Federal Fluminense, Niteroi, Brazil; 6 Excellion Biomedical Services, Petrópolis, Rio de Janeiro, Brazil; 7 National Institute of Metrology, Quality and Technology - INMETRO, Xerém, Rio de Janeiro, Brazil; 8 Department of Biomaterials and Biomimetics/Director for Research Department of Periodontology and Implant Dentistry, New York University College of Dentistry, New York, New York, United States of America; Université de Technologie de Compiègne, France

## Abstract

**Background:**

It is known that physico/chemical alterations on biomaterial surfaces have the capability to modulate cellular behavior, affecting early tissue repair. Such surface modifications are aimed to improve early healing response and, clinically, offer the possibility to shorten the time from implant placement to functional loading. Since FAK and Src are intracellular proteins able to predict the quality of osteoblast adhesion, this study evaluated the osteoblast behavior in response to nanometer scale titanium surface texturing by monitoring FAK and Src phosphorylations.

**Methodology:**

Four engineered titanium surfaces were used for the study: machined (M), dual acid-etched (DAA), resorbable media microblasted and acid-etched (MBAA), and acid-etch microblasted (AAMB). Surfaces were characterized by scanning electron microscopy, interferometry, atomic force microscopy, x-ray photoelectron spectroscopy and energy dispersive X-ray spectroscopy. Thereafter, those 4 samples were used to evaluate their cytotoxicity and interference on FAK and Src phosphorylations. Both Src and FAK were investigated by using specific antibody against specific phosphorylation sites.

**Principal Findings:**

The results showed that both FAK and Src activations were differently modulated as a function of titanium surfaces physico/chemical configuration and protein adsorption.

**Conclusions:**

It can be suggested that signaling pathways involving both FAK and Src could provide biomarkers to predict osteoblast adhesion onto different surfaces.

## Introduction

Endosseous implants are widely used for the restoration of edentulism with long-term success rates often exceeding 90% [1]–[3]. This is one of the most successful treatment modalities in the field, and has significantly improved the patients' quality of life. In order to further improve treatment success rate, different levels of modifications of implants have been emphasized. Among those, surface modification has been extensively investigated provided that it is the first component to interact with the host [4]. For instance, the so-called moderately micro roughened surface, with the arithmetic average height deviation (S_a_) of approximately 1.5 µm, was shown to present enhanced bone response relative to turned or excessively roughened surfaces [5]–[8].

Recent research has suggested that the presence of nanotopography may be one of the decisive factors for early osseointegration [9], [10]. Surface modification at the nanolevel was shown to increase the bioactivity of the implant surface, which resulted in significant enhancement of new bone formation *in vivo* [11], [12]. Of great interest was the fact that the nanotextured surfaces not only enhanced the bone formation but also strengthened the biomechanical properties [13], [14]. Also of relevance are nanochemical alterations involving hydroxyapatite or other calcium phosphate compositions (CaP). *In vivo* studies have shown that the application of nanometer scale CaP coatings has contributed in improving the early bone response in both histomorphometric analysis and torque to interface fracture mechanical testing [15], [16]. Furthermore, the effect of calcium and phosphate seemed to have improved mineralization of the bone surrounding the implant. It has also been reported that nanoscale CaP applied to titanium alloy implant surfaces significantly improved the nanomechanical properties of the interfacial bone [10]. Moreover, it has been confirmed that genes responsible for active bone mineralization were significantly upregulated for the nanoscale CaP coated implants compared to a non-coated implant surface [17].

Although the phenomenological results suggest that the modification at the nanoscale is an enhancing factor for osseointegration, the detailed interfacial interactions between the nanostructures and the osteogenic cells have not been clarified to a full extent. It is difficult to illustrate the detailed biologic events in the cellular/molecular level with only the *in vivo* experimental approaches. Thus, understanding the *in vitro* implant surface/cellular interactions could potentially provide better interpretations of the *in vivo* biologic osseointegration cascade.

It has been suggested that the recruitment/migration of cells to the implant surface is one of the most important events for an enhanced osseointegration [4]. The ability to better adhere and spread the recruited cells to the implant surface has been proven to be an essential factor for the subsequent osteogenic events [18], [19]. It has been shown *in vitro* that nanotextured surfaces influence the cell morphology of the adhered osteoblasts [20], [26]. Furthermore, Zhang *et al*. has reported that nanostructures superimposed in the microrough structure significantly enhanced the cell adhesion and cell spreading, which was confirmed by an actin immunofluorescent staining [21]. Thus, it is quite evident from these morphological reports that nanostructures do have an influence on the initial biomaterial-cellular interactions, mainly governed by a well-orchestrated phosphorylation cascade within the cells.

In order to further clarify these interactions, understanding the involved signaling events are essential. We have focused on investigating the molecular mechanism that governs osteoblast adhesion on different surfaces in an attempt to establish a map of signaling proteins able to guide the development of novel biomedical materials [22], [23]. As a result, it was identified that transient focal adhesion kinase (FAK) and Src activations are involved in the cell signaling upon the integrin activation and suggested that these proteins could be explored as biomarkers of cell-biomaterial interface [24]. Therefore, the aims of this *in vitro* study were to analyze the activation of FAK and Src during the earlier cellular adaptation on 4 different engineered titanium surfaces, and to determine whether the nanoscale surface modification has an impact on the activation of the proposed signaling proteins.

## Material and Methods

### Materials

Four different implant surfaces (n = 5, each) were used for the study: machined (M), dual acid-etched (DAA), resorbable media microblasted and acid-etched (MBAA), and acid-etch microblasted (AAMB) (Ossean, Intra-Lock International, Boca Raton, FL, USA). All materials were sterilized by exposure to Gamma irradiation. Antibodies: Anti-phospho-Src (Y416), anti-Src, anti-phospho-FAK (Y397), anti-phospho-FAK (Y925), anti-FAK, anti-CDK 6, anti-beta-actin were used (Cell Signaling Technology, Inc., Danvers, MA, USA).

### Surface characterization

Surface topography at the nano level was characterized by means of an atomic force microscope (AFM, XE-100, Park Systems, Suwon, Korea). Three randomly selected discs were used, and three regions on each disc were measured using a non-contact mode setup in air and at room temperature (scan size 1×1 µm). The parametric calculation was performed after the removal of errors of form and waviness by the use of a Gaussian filter (0.25×0.25 µm).

Micro level surface topography was evaluated by an optical interferometer (IFM,MicroXam; ADE Phase Shift, Inc., Tucson, AZ, USA). The same discs used in the AFM were used and three randomly selected regions were selected per disc. The parametric calculation was performed after the removal of errors of form and waviness by the use of a Gaussian filter (50×50 µm).

For both evaluations, the following 3D parameters were selected: Sa (i.e. arithmetic average height deviation from a mean plane), Sds (i.e. density of summits), and Sdr (i.e. developed surface ratio). Descriptive 3D images were reconstructed with imaging software MountainsMap 6.2 (Digital Surf, Paris, France).

Surface morphology of the implants was examined by scanning electron microscopy (SEM) using a LEO Ultra 55 FEG (Zeiss, Oberkochen, Germany) at an accelerating voltage of 5 kV. A secondary electron detector was used to acquire the images. Three randomly selected implants from each group were investigated.

Confirmation of the chemical composition was performed using energy dispersive X-ray spectroscopy (EDX) through an Inca system (Oxford Instruments, Oxfordshire, UK) connected to the SEM. For the Energy Dispersive Spectrometer (EDS) analysis, an accelerating voltage of 10 kV was used and three randomly selected parts on each surface were analyzed at a magnification of 1 kX.

### Cell culture

MC3T3-E1 (ATCC 7594) mouse pre-osteoblastics cells were used in this study. Cells were cultured in alfa-MEM supplemented with 10% of fetal bovine serum (FBS) at 37°C and 5% CO2. Co-confluent passages were tripsinized and used in all experiments in 24 well plates.

### Cytotoxicity assay

Samples were prepared according to ISO10993-12:2008. Briefly, surface treated sterile discs of titanium were transferred to Petri dishes containing culture media (α-MEM) on a ratio of 1 mL per 6 cm^2^ of tested material. After 24 h incubation at 37°C, extracts were collected and tested for cytotoxicity, as follows. High density Polystyrene beads extract and 2% Phenol were used as negative and positive controls, respectively. The cells were then sub-cultured on 96-well plates at a cell density of 8, 5×10^3^ cells/cm^2^. After incubation for 24 h, the culture media was completely removed and substituted by 180 µL/well of each sample extract (n = 5) plus 20 µL 10% FBS. An internal control was assayed by keeping the cells exposed solely to supplemented culture medium. After 24 h exposure, the cytotoxic potential of each sample (extracts) or controls were evaluated by a multiparametric assay (In Cytotox, Xenometrix, Germany), in which three different parameters of cell viability are simultaneously tested using the same sample, such as: mitochondrial activity (XTT-test), membrane integrity (NR test) and cell density (CVDE test). The XTT-test measures mitochondrial dehydrogenase activity by the conversion of the yellow water-soluble tetrazolium salt XTT into orange-colored soluble compound of formazan, by measuring in absorbance at 480 nm (Synergy II; BioTek Instruments, USA). Thus, the production of formazan indirectly indicated the viability of cells. NR test measures the capacity of membrane-intact cells to incorporate and accumulate the neutral red dye on its lysosomes, which can be detected after extraction by its absorbance at 540 nm. Lastly, Crystal violet dye elution (CVDE) test was carried out, based on nuclear protein and DNA staining, followed by exhaustive washing, dye extraction and evaluation of the relative cell density by absorbance measurement at 540 nm.

### Western blotting

Cells were cultured on different surfaces and after 3 hours they were removed out and protein extracts were obtained using a Lysis Cocktail (50 mM Tris [tris(hydroxymethyl)aminomethane]–HCl [pH 7.4], 1% Tween 20, 0.25% sodium deoxycholate, 150 mM NaCl, 1 mM EGTA (ethylene glycol tetraacetic acid), 1 mM O-Vanadate, 1 mM NaF, and protease inhibitors [1 µg/mL aprotinin, 10 µg/mL leupeptin, and 1 mM 4-(2-amino-ethyl)-benzolsulfonyl-fluorid-hydrochloride]) for 2 h on ice. After clearing by centrifugation, the protein concentration was determined using Lowry method. An equal volume of 2x sodium dodecyl sulfate (SDS) gel loading buffer (100 mM Tris-HCl [pH 6.8], 200 mM dithiothreitol [DTT], 4% SDS, 0.1% bromophenol blue, and 20% glycerol) was added to samples and boiled for 5 minutes. Proteins extracts were resolved by SDS-PAGE (10 or 12%) and transferred to PVDF membranes (Bio-Rad, Hercules, CA, USA). Membranes were blocked with either 1% fat-free dried milk or bovine serum albumin (2.5%) in Tris-buffered saline (TBS)–Tween 20 (0.05%) and incubated overnight at 4°C with appropriate primary antibody at 1∶1000 dilutions. After washing in TBS-Tween 20 (0.05%), membranes were incubated with horseradish peroxidase-conjugated anti-rabbit, anti-goat or anti-mouse IgGs antibodies, at 1∶2000 dilutions (in all immunoblotting assays), in blocking buffer for 1 hour. Thereafter, the detection was performed by using enhanced chemiluminescence (ECL).

### Alkaline Phosphatase (ALP) activity determination

3 mL of MC3T3-E1 pre-osteoblastic cells in suspension (75×10^3^ cells/mL) were seeded in 6-wells dish plate (polystyrene group) or Ti-discs (test's groups) until 85–90% of confluence. Thereafter, the cells were stimulated to differentiate by remaining then under differentiation medium containing ascorbic acid (50 µg/mL) and β-glycerophosphate (10 mM) during 7 and 14 days. After, the adherent cells were rinsed with ice-cold PBS and incubated for 30 minutes at room temperature with ALP assay buffer (100 mM of Tris-HCl, pH 9.0, 1 mM of MgCl_2_) containing 1% Triton X-100. The cell extracts were removed from dishes, centrifuged, and used for the enzyme assay. The ALP activity was determined using 5 mM of pNPP as substrate. One unit of enzyme activity was defined as the amount of enzyme that converted one µmol of substrate to product per minute. Protein concentrations were determined by the Lowry method. The relative percentages of data were normalized from mean of polystyrene at 7 days (used as a internal control).

### Proteomics analysis of adsorbed serum proteins on Ti surfaces

Proteins were extracted from titanium discs. Briefly, three titanium disks from each condition were incubated with 5 mL of DMEM supplemented with 10% bovine serum for 3 h at 37°C in 5% CO2, rinsed with PBS twice and incubated at room temperature for 20 min with 1 mL of extraction buffer solution (8 M urea, 10 mM DTT, 100 mM Tris pH 8.6). Solution was removed with the aid of a cell scraper, pooled, washed with 50 mM NH4CO3 and concentrated to a final volume of 50 µL using a 3 kDa cutoff Amicon centrifugal filter (Millipore). Samples were then prepared and digested with trypsin and identified by LC/MS in a nanoacquity nanoUPLC system coupled to a Synapt G1 Q-TOF mass spectrometer (Waters, USA). Data was processed for identification and quantification of proteins using Proteinlynx Global Server v2.5.

### Statistical analysis

Mean values and standard deviation obtained for each test were calculated, and one-way ANOVA was performed (alpha error type set to 0.05), with Bonferroni corrected post-test, using GraphPad Prysm 5 (GraphPad Software, USA).

## Results

### Sample characterization

The results from the AFM and IFM measurements are presented in **Table 1**. In brief, the surface topography (in terms of roughness parameters) at the micro-level evaluated by IFM presented no statistical differences between the AAMB, MBAA, and DAA groups but for one parameter, where Sdr was significantly higher than other groups. The M surface presented the lowest values for all parameters as compared to the other groups. The surface topography at the nano-level as measured by AFM presented characteristics differing from those at the micro-level. Interestingly, AAMB and MBAA presented significantly higher values in terms of the height parameter Sa, which suggests that these surfaces were modified at the nano-level, regardless of their similarity at the micro-level as revealed by IFM.

**Table 1:** The results from the AFM and IFM measurements.

AFM	Sa (nm)	Sds [1/µm2)	Sdr (%)
**AAMB**	7,16	2060,44	18,89
**MBAA**	6,73	1361,00	16,50
**DAA**	2,86	570,29	22,82
**M**	2,76	2277,67	3,70

IFM	Sa (µm)	Sds [1/µm2)	Sdr (%)
**AAMB**	0,99	0,07	22,67
**MBAA**	1,00	0,06	24,53
**DAA**	1,08	0,08	52,79
**M**	0,18	0,10	1,56

**Figure 1:**
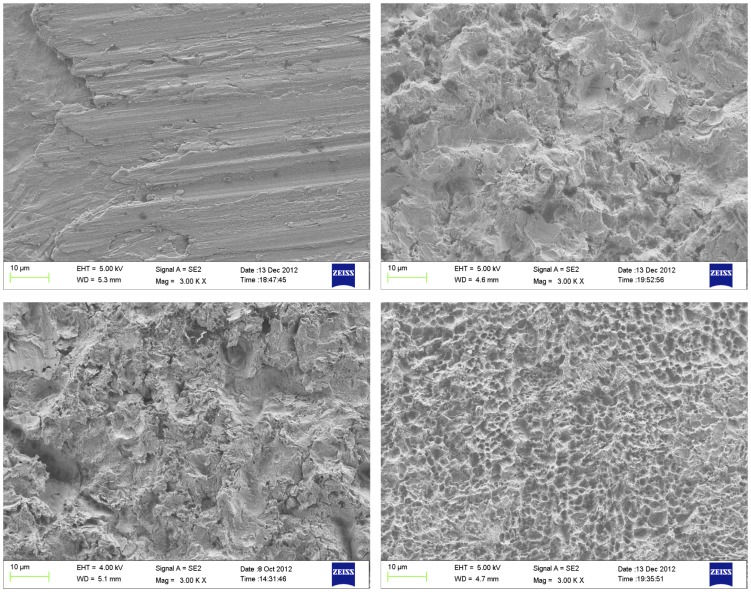
SEM micrographs taken at 3 kX magnification of (top left) M, (top right) AAMB, (bottom left), MBAA, (bottom right) DAA.

**Figure 2:**
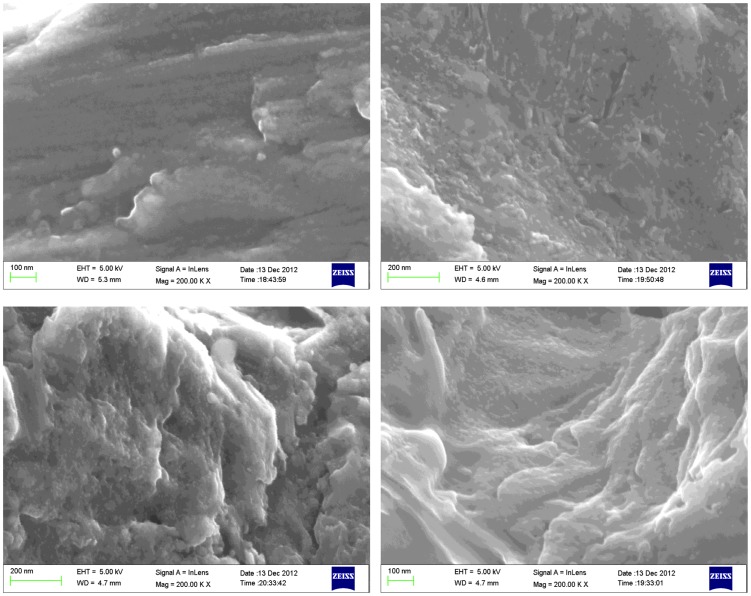
SEM micrographs taken at 200 kX magnification of (top left) M, (top right) AAMB, (bottom left) MBAA, and (bottom right) dual acid etched.

SEM micrographs obtained at two different magnifications (3 kX and 200 kX) for the investigated surfaces are shown in **Figures 1** and **2**. Qualitative differences between the surface treatments could be seen, especially at the lower magnification (**Figure 1**). The machined surface (M) appears smoother than the others and the dual acid etched has the highest porosity, while the MBAA and AAMB surfaces presented similar rougher and less porous features. At higher magnifications (**Figure 2**), the differences are less obvious. However, more nano-sized features seem to be present on the MBAA and AAMB surfaces in comparison to the M and DAA. Once again, no evident differences could be distinguished between the MBAA and AAMB surfaces.

Elemental chemical composition evaluation through EDS is presented in **Table 2**. For all the titanium-based samples, the highest elemental amounts were observed for Ti, followed by C and O. Trace amounts of P and Ca were detected on the DAA and MBAA surfaces, while F was detected on the AAMB surface. Sodium was only detected for the DAA surface.

**Table 2:** Elemental chemical composition through EDS.

Atomic %	F	Al	P	Ca	N	Ti	C	V	O	Na
**Polystyrene**							98.3 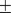 1.2		trace	trace
**M**						88.2 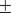 1.4	6.3 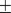 0.6		5.6 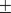 0.9	
**DAA**			0.2 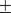 0.1	trace		76.5 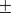 3.2	10.3 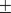 2.0		12.8 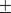 1.1	0.2 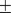 0.1
**MBAA**			0.4 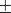 0.02	0.3 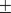 0.03		63.6 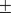 0.9	17.1 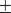 0.7		18.7 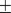 0.3	
**AAMB**	2.7 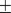 0.2					75.6 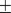 3.4	11.1 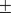 0.7		10.6 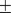 2.8	
										

### Different modifications on Ti-surface did affect osteoblast cytocompatibility

**Figure 3:**
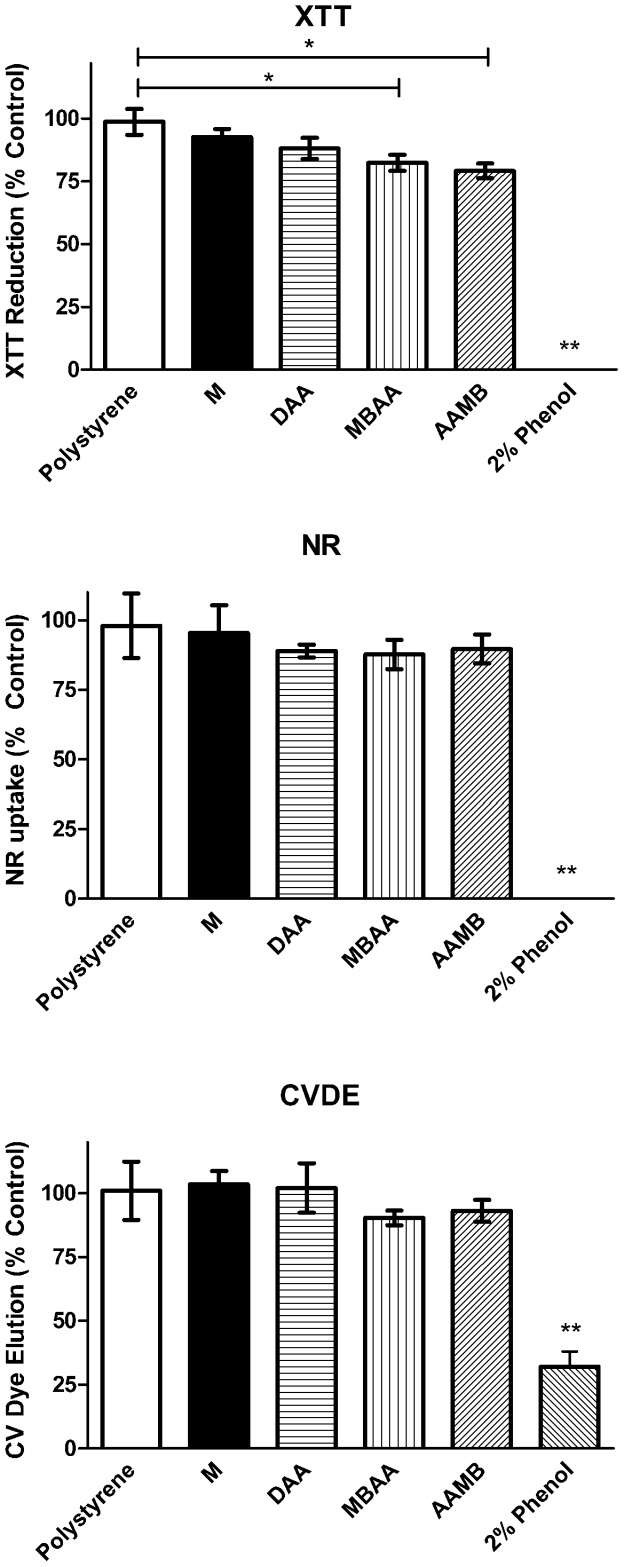
Multiparametric cytotoxicity assay. Cytotoxic effects of extracts of Titanium Discs with different treatments, as measured by mitochondrial dehydrogenase activity (XTT assay), membrane integrity (NR: Neutral Red assay) or density of cells (CVDE: Crystal Violet Dye Elution Assay), and represented as a percentage of control (tissue plastic) cell viability. *Statistically significant differences between groups (p<0.01). ** Statistically different from all other groups (p<0.001). 2% Phenol and Polystyrene were used as positive and negative controls for cytotoxicity, respectively.

In this study, to determine the viability of pre-osteoblast cells we accessed 3 independent parameters: metabolism, membrane integrity and viable cell density. Briefly, we kept different surfaces in culture media for 24 hours. Thereafter, we used those media to evaluate its cytotoxicity in osteoblasts. This sequence was chosen since it concentrates the substances released from the different materials for 24 hours. **Figure 3** shows that no cytotoxic effects were observed in osteoblasts *in vitro* when cultured in the presence of conditioned media from the different material surfaces. Therefore, we may assume that no difference observed on the subsequent experiments would be related to impairment of cell viability due to relevant changes induced by the different Ti surfaces on the culture media.

### Nano-topographical changes modulate cell proliferation by affecting CDK6 expression

**Figure 4:**
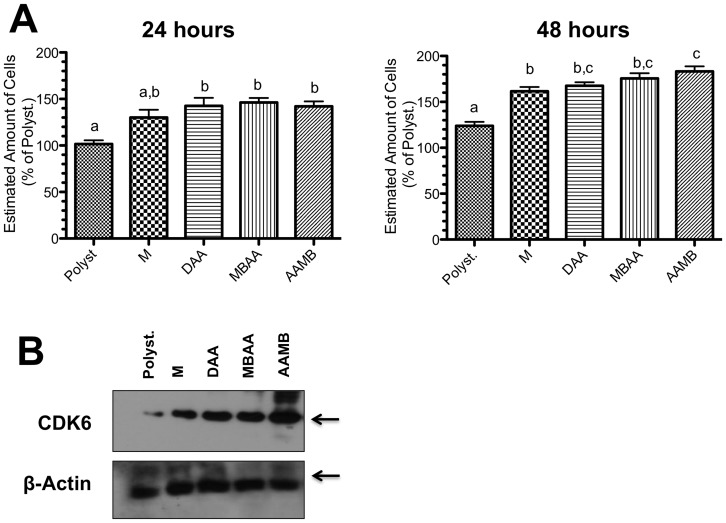
Ti-modified surfaces promote osteoblast proliferation. The cells were cultured on the different kind of Ti-modified surfaces in order to estimate cellular proliferation. **A**) Graph shows cell growth at 24 and 48 hours. Cells were cultured on different titanium discs and thereafter stained with crystal violet, as detailed in material and methods. **B**) Cells were cultured on Titanium surfaces and after 48 hours they were lysed in order to analyze CDK6 expression by performing western blotting approach. The results showed that Ti surfaces are able to stimulate CDK6 expression, a signaling protein known to control cell cycle progression, and which has been associated to conditions of increased proliferation of mouse osteoblasts. In graphs (A), letters mean significant differences (ANOVA with Bonferroni corrected post-test).

In order to identify osteoblast behavior, their capacity of adhering and proliferating onto the nanometric scaled titanium surfaces was evaluated. Thus, osteoblasts were seeded by following classic protocols and the biological sample was obtained up to 48 hours from seeding. Firstly, we identified that osteoblast proliferating profile was dependent on Ti-surface, respecting nanometric scaled surfaces and statistical significance was found on both 24 and 48 hours from seeding (*letters*, **Figure 4A**). The results showed that the different surfaces yielded profound changes in osteoblast behavior, at least in affecting the molecular machinery involved with cellular proliferation. All textured titanium surfaces promoted an increase of CDK6 expression at 48 hours from seeding when compared with the M and polystyrene (control) surfaces (**Figure 4B**). However, no significant changes were observed between Ti-modified surfaces.

### Src and FAK detected suitable Ti-nanoscaled surfaces

FAK and Src have been proposed as biosensors of eukaryotic cells adhesion mainly upon integrin activation. Over the last years we have applied Src and FAK detection in an attempt to predict the biological quality of cell/material interactions.

**Figure 5:**
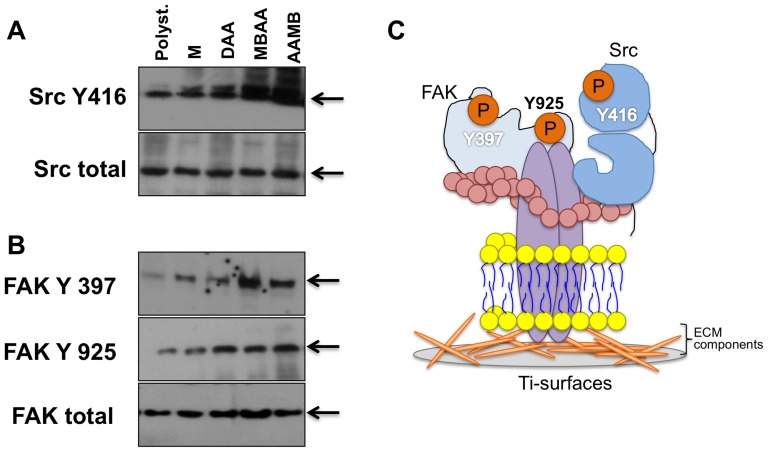
Signaling proteins during pre-osteoblast attachment on nanometer scale titanium surface texturing. To assess signaling proteins involved in initial osteoblast response on titanium discs, we checked the phosphorylation profile of proteins related to focal adhesion components [**A**: Src (Y416) and **B**: FAK (Y925, Y397)]. In all experiments, the pre-osteoblasts cells were seeded on different Ti-surfaces or polystyrene and after 3 h the samples were collected to perform western blotting approach. Briefly, the results showed that there is a balance of phospho-proteins, at Y-residue, in response to nanoscaled Ti-surfaces. However both FAK and Src have been activated by all Ti-surfaces evaluated in this study, it was clear that MBAA provoked a bigger phosphorylation of FAK at Y397 when compared with others; the same phosphorylation profile was found to Src; **C**) Schematization of signaling proteins proposed in this work. Upon integrin activation e Integrin binding results in the recruitment of focal adhesion kinase (FAK), a cytoplasmic protein-tyrosine kinase. Focal adhesions are often the most prominent sites of tyrosine phosphorylation in eukaryotic cells, and FAK is one of the major tyrosine-phosphorylated proteins found at these sites. The clustered FAK molecules cross-phos- phorylate each other on a specific tyrosine (Y), thereby creating a phosphotyrosine docking site for members of the Src family of cytoplasmic tyrosine kinases. These kinases then phosphorylate FAK on additional tyrosines, creating docking sites for a variety of intracellular signaling proteins; they also phosphorylate other proteins in focal adhesions. In general, these intracellular events will culminate to cell morphological changes favoring adhesion, migration or differentiation by rearranging their cytoskeleton molecules.

Our results showed that surface topography at the nanometer scale resulted in a specific modulation of FAK and Src phosphorylation (**Figure 5**). Both phosphorylations were up-regulated in response to surface modification. The phosphorylation profiles were similar among the groups but a particular increase in response to MBAA surfaces was observed regarding FAK phosphorylation (**Figure 5B**), while Src remained unchanged (**Figure 5A**). It is worth noting that we evaluated 2 different sites of phosphorylation on FAK: Y397 and Y925. Also, it was observed that Src phosphorylation at Y416 was increased in response to nanotopographical changes, being more evident for both MBAA and AAMB surfaces. In summary, the MBAA surface modification highly modulates FAK and Src phosphorylation, likely resulting in osteoblast adhesion (**Figure 5C**).

### Modified Ti substrates allow osteoblast differentiation

**Figure 6:**
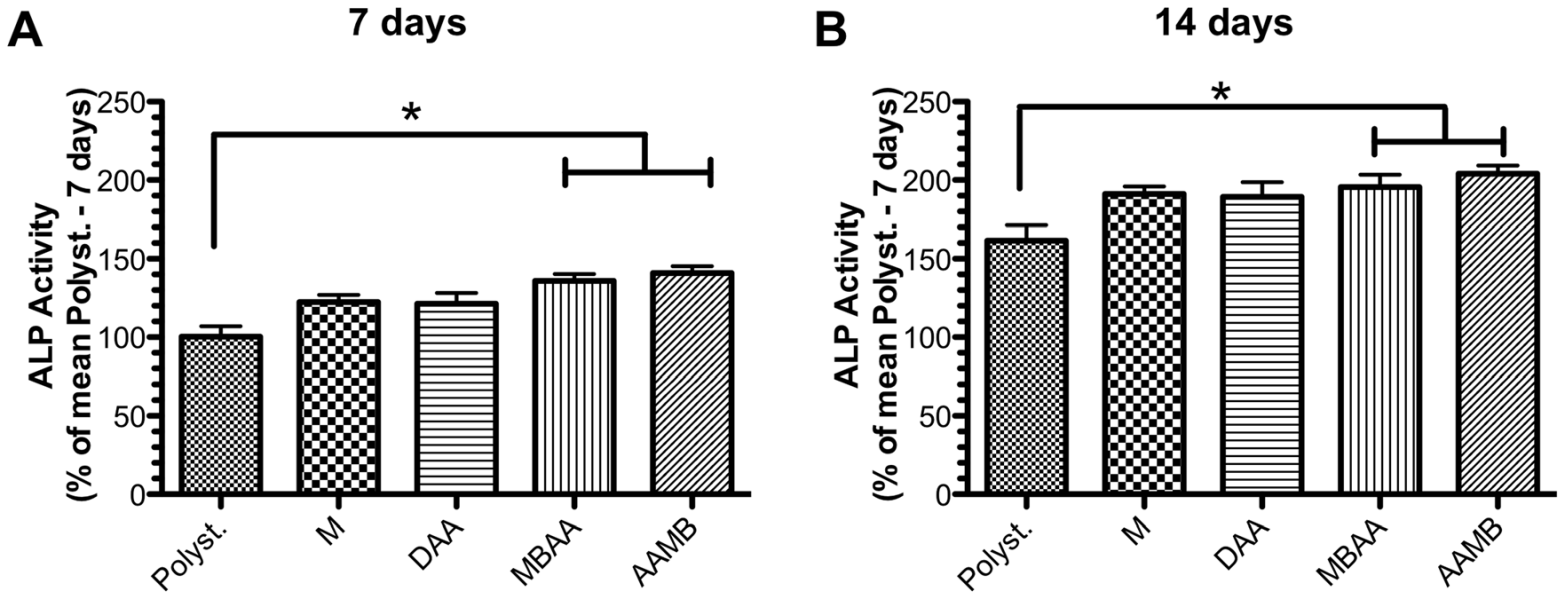
Different Ti-based surfaces are able to promote preosteoblast differentiation. Preosteoblasts were seeded on discs of Ti for 7 (**A**) and 14 days (**B**). Afterward, the samples were collected, and alkaline phosphatase (ALP) activity was measured using *p*NPP as substrate. Our data showed that all surfaces tested were able to guarantee osteoblast differentiation, cellular mechanisms expected to promote implant osseointegration. It is important to mention that the relative percentages of data were normalized from mean of polystyrene at 7 days. Significant differences were considered when p<0.05 (*, ANOVA with Bonferroni corrected post-test).

To investigate the influence of modified Ti substrates on osteoblast differentiation, ALP activity was evaluated. As previously described in the literature, ALP activity is a parameter often used to identify early osteoblast differentiation [25]. The protocol used in this work could estimate ALP activity by measuring the formation of *p*-nitrophenol from catalysis reaction of *p*-nitrophenilphosphate by ALP *in situ*. **Figure 6** shows the ALP activities of pre-osteoblastic cells cultured on the different Ti surfaces after 7 and 14 days. It was shown that all surfaces evaluated (M, DAA, AAMB, MBAA) were able to promote osteoblast differentiation, it being that AAMB and MBAA provoked a significant increase in ALP activity [considered when p<0.05 (*)] in both 7 and 14 days.

### Adsorbed proteins identification and quantification

**Figure 7:**
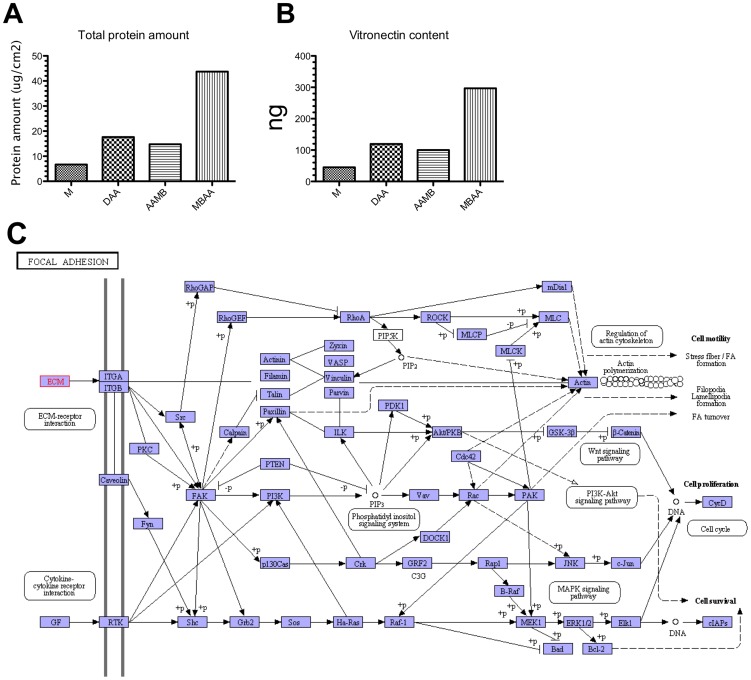
Protein adsorption onto different Ti-surfaces. The Ti-discs were incubated in cell medium containing 10% bovine serum. The adsorbed proteins were extracted from titanium discs and the samples then prepared and digested with trypsin. Then, identified by LC/MS in a nanoacquity nanoUPLC system coupled toa Synapt G1 Q-TOF mass spectrometer. **A**) The total protein adsorption at per square centimeter; **B**) Relative amount of vitronectin: in this regard, it was possible to verify that MBAA's surface properties provided a better adsorption of vitronectin when it was compared with others; **C**) Graphical presentation of focal adhesion pathway obtained from KEGG database. Considering the role of vitronectin on osteoblast adhesion, it is possible that the impact of these nanosurfaces on cell adhesion may be mediated by protein adsorption.

In order to identify if the different surfaces could adsorb potential ligands that could trigger cellular adhesion pathways, a proteomic analysis of absorbed serum proteins on the implants surfaces was performed. A total of 18, 33, 32 and 64 different proteins were identified in the M, MBAA, AAMB and DAA conditions respectively, of which 7, 16, 15, 26 were respectively quantified (see Data S1). The total amount of proteins (µg)/cm2 was higher on MBAA, when compared to the others surfaces (**Figure 7A**). Using the Panther Classification system (http://www.pantherdb.org/), it was possible to identify 8, 9, 9 and 10 biological processes for M, MBAA, AAMB and DAA, respectively, from which vitronectin could be identified as a protein involved in biological adhesion (GO:0022610, see scheme **Figure 7C**). In this regard, it was possible to verify that MBAA's surface properties provided a better adsorption of vitronectin as compared with other surfaces (**Figure 7B**).

## Discussion

The adhesion of osteogenic cells to implant surfaces occurs subsequently to the extra cellular matrix assembly. Thus, exploring the molecular mechanisms related to this phenomenon in detail may clarify the role of nanotextured surfaces. Biological evaluation of surfaces using cell culture has emerged as an alternative to *in vivo* models in an attempt to predict the biocompatibility of novel biomaterials [28], [29]. These studies in general consider cell viability, adhesion, proliferation, and differentiation of different types of cells and such methodology has become the standard for initial material biocompatibility screening. While valuable information has been extracted from cell culture studies with regards to cell adhesion to titanium substrates with different topographies, little information is usually obtained concerning the signaling pathways and their influence on cell adhesion and spreading [22], [30].

The present study evaluated how nanochemically modified surfaces affected the pathway for osteoblastic cell adhesion. The surface topographic analysis showed that all of the groups tested possessed different surface topographies. When evaluated at the micrometer level length scale, no differences were detected between the MBAA, AAMB, and DAA surfaces. However, when analysis was performed at the nanometer length scale, MBAA and AAMB presented higher degrees of texture. While the MBAA and AAMB presented similar S_a_ values at the nanometer scale, the AAMB surface presented significantly higher S_ds_ values (density of summits) than the MBAA groups, which suggests that the peeks (summits) are differently distributed due to the different processing methods. This effect may be attributed to the fluoride detected in the EDS evaluation since there are reports indicating that the presence of fluoride or treatment with fluoride in fact alters the surface topography in the nano-level [12], [20], [27]. Although it is difficult to directly correlate the differences in surface topography and chemistry to the cellular events, it can be speculated that the obtained outcomes were greatly influenced by the different surface modifications.

In this study, we utilized a sequence of analysis to evaluate different titanium surfaces through parameters of cytotoxicity (MTT, XTT, and NR), osteoblast adhesion, proliferation, and differentiation. The initial cell toxicity evaluation showed that all the different surface treatments did not result in cytotoxic outcomes, and that biological effects should be more related to surface contact than to alteration on culture media content. The subsequent method evaluated osteoblast behavior on the different surfaces for up to 48 hours. This time point was selected due to the possibility of identifying the proteins involved in the cell cycle progression. In this case, it was found that textured titanium surfaces resulted in an increase in the osteoblast number compared to the machined and polystyrene surfaces, which was in accordance with previous investigations [31], [32]. Curiously, our results showed that textured titanium surfaces stimulated CDK6 expression, a signaling protein known to control cell cycle progression, and which has been associated to conditions of increased proliferation of mouse osteoblasts [33], [34] However, different effects for diverse nanotopographies were not evident, suggesting that this may be a general feature of nanostructured surfaces.

FAK and Src phosphorylation were evaluated in response to different surfaces and it was observed that both markers are main signaling proteins during osteoblast adhesion to the textured titanium surfaces. Furthermore, FAK phosphorylation at Y397 profile (autophosphorylation) was more evident in response to the nanostructured MBAA relative to the other surfaces, while Src remained active in all groups. From these evidences, we can conclude that chemical/morphological properties of surfaces define cell behavior.

The present results also provided a possible explanation for the mechanism in which nanotopography may affect such processes, as revealed by the proteomic analysis performed on the surfaces after exposure to serum. The profile of protein adsorption varied with each nano-surface on both total protein content and the type of preferentially adsorbed proteins. This result becomes relevant on the light of the well-known role of Extracellular Matrix proteins on osteoblast adhesion mediated by integrin on biomaterial surfaces including functionalized Ti [35]. Furthermore, the content of vitronectin was highly increased on the group with higher levels of FAK phosphorylation (MBAA). Considering the role of vitronectin on osteoblast adhesion [36], it is possible that the impact of these nanosurfaces on cell adhesion may be mediated by protein adsorption. It is important to note that increased vitronectin contents are able to induce the phosphorylation of focal contact proteins [37] on osteoblasts.

Finally, the evaluation of osteoblast differentiation showed that all surfaces stimulate osteoblast differentiation by promoting alkaline phosphatase activity (ALP). Once again, only the two more nano-roughed surfaces (AAMB and MBAA) were able to induce a statistically significant increase on ALP activity as compared to polystyrene (p<0.05). As previously demonstrated, ALP is an accepted biological parameter to determine osteoblast differentiation [38], [39]. This suggests that pre-osteoblast adhered onto those nano-structured surfaces were able to differentiate and produce extracellular components, an essential cellular event for improving the bone mass and for integrating materials to host hard tissue.

Although the study of signaling pathways provided further information on the effect of nanochemical modifications, the overall outcomes were comparable for all textured surfaces. However, the fact that FAK phosphorylation was significant for the nanotextured MBAA surface suggests that signaling pathways involving FAK may provide important markers for the recognition of interesting nanoscale structures for cell adhesion.

## Supporting Information

Data S1 (XLS)
